# Abstracts from Foreign Journals

**Published:** 1901-11

**Authors:** M. P. Ravenel, S. J. J. Harger

**Affiliations:** Philadelphia, Pa.; Philadelphia, Pa.


### ABSTRACTS FROM FOREIGN JOURNALS.

### FRENCH.

Under the Direction of M. P. Ravenel, M.D., and S. J. J. Harger, V.M.D., of Philadelphia, Pa.

Hypothermia. Cantiget reports two cases. The first was an ox, which suffered from violent abdominal pain. The temperature was 35° C. for twenty-four hours, after which recovery followed.

The second case was an ox suspected of tuberculosis; temperature 38.6° C. Injected tuberculin. The temperature then fell to 36° C. and remained so during the test. In Nocard's opinion, tuberculosis was present, but no autopsy was made.

Bouchet observed a third case of hypothermia in a donkey which had been castrated and clipped at the beginning of winter. The temperature descended to 34.4° C. in spite of all means employed, and remained so until spring. The cause assigned for this phenomenon was the suppression of the internal secretion of the testicles.—*Ann. de Med. Vet.*

Testing Crude and Boiled Milk. This test determines whether or not milk has been heated to a temperature of 80° C. or more, as in the process of sterilization or in the question of milk from tuberculous cows which it may be the custom to feed to pigs and calves only after boiling. To raw milk add 10 per cent. of tincture of guaiacum wood, agitate, and, if necessary, heat, but below 80° C. The mixture rapidly becomes blue, which then disappears slowly; with boiled milk, to which crude milk has been added, the phenomena are the same; when, on the contrary, milk has been heated above 80° C. the mixture will be of a dirty yellow instead of a blue coloration. This test may be used in certain legal aspects when milk must be sold after having passed through certain processes.—*Annales de Med. Vet.*

Polynuclear Hyperleucocytosis in Diagnosing Liver Abscess. According to Boinet, the examination of the blood of individuals affected with large abscesses of the liver shows a large quantity of leucocytes. Their proportion is from six to ten times that of the normal, and in two cases the author counted forty white blood-cells in one field of the microscope under 420 diameters. Staining with methylene blue or thionin reveals numerous leucocytes with several nuclei.

This leucocytosis is more pronounced in abscess of the liver than in other forms of suppuration. It is analogous to the "leukæmia of suppuration" indicated by Malassy in renal abscess, suppurative pleurisy, cold and acute abscess, etc. Hypernuclear leucocytosis is, therefore, an important element in diagnosing abscess of the liver.—*Annales de Med. Vet.*

COCAINE ANÆSTHESIA OF THE SPINAL CORD. Cuillé and Sandrail have employed this method of anæsthesia in veterinary surgery. In the horse, ass, and ox the spinal puncture is made with a trocar and canula, 0.10 in. long and 0.0015 m. in diameter. The puncture is made vertically on the median plane of the body on a line uniting the two internal angles of the skilia. This direction leads into the lumbosacral space, and is the method selected in all the domestic species. Disinfection of the skin, sterilization of the needle and the solution injected must be absolute. For the dog the needle of a hypodermic syringe suffices, the animal being held lying on the side.

*Operations Practised.* Horse and ass, 5: Nerve resections of hind limb, ovariotomy, point-firing. Injection, 4 c.c., 2 c.c., and 1 c.c., respectively, of a 2 per cent. solution. Result, complete insensibility of the member except in the case of firing, the animal slightly moving the member from time to time. Cattle, 3: Deep punctures at different points of the hind-quarters and firing of stifle. Respective injections, 5 c.c. and 1 c.c. of 2 per cent. solution. Results like preceding. Dogs, 4: Punctures and cauterization of stifle; injection, 1 c.c. and 2 c.c.

One of the dogs after recovering received 1 c.c. of the solution into the atlo-axoid space without puncturing the spinal cord. In a few moments the animal crouched down and was able only to crawl along, the sternum dragging on the ground; vomiting. The anæsthesia now also involved the anterior part of the body, excepting the head. He recovered in a few hours.

These experiments of Cuillé and Sandrail, although few, tend to show that cocaine anæsthesia of the spinal cord presents no danger, and may be of value in surgical operations of the posterior extremities. These observers, in such cases, consider the practice justified.—*Annales de Med. Vet.*

